# Comparative Study of Antioxidant Power, Polyphenols, Flavonoids and Betacyanins of the Peel and Pulp of Three Tunisian *Opuntia* Forms

**DOI:** 10.3390/antiox2020037

**Published:** 2013-04-19

**Authors:** Nizar Yeddes, Jamila K. Chérif, Sylvain Guyot, Hélène Sotin, Malika T. Ayadi

**Affiliations:** 1 Laboratory Applications of Chemical Resources, Natural Substances and the Environment (LACReSNE), Faculty of Sciences of Bizerte, University of Carthage, 7021 Zarzouna-Bizerte, Tunisia; E-Mails: cherif_j2000@yahoo.fr (J.K.C.); malikatrabelsi_ayadi@yahoo.fr (M.T.A.); 2 Tunis Preparatory Institute for Engineering Studies (IPEIT), Montfleury, University of Tunis, 1008 Tunis, Tunisia; 3 INRA, Research Unit Cider, Biotransformation of Fruits and Vegetables (URCBFL), B.P. 35327, F-35653, Rennes Le Rheu, France; E-Mails: Sylvain.Guyot@rennes.inra.fr (S.G.); helene.sotin@rennes.inra.fr (H.S.)

**Keywords:** *Opuntia ficus indica*, *Opuntia stricta*, cactus fruit, antioxidant activity, phenolic compounds

## Abstract

The antioxidant activity and the chemical composition of methanol extracts from peel and pulp belonging to two species of Tunisian prickly pears *Opuntia ficus indica* (spiny and thornless forms) and *Opuntia stricta* have been studied. The antioxidant capacity was measured by DPPH radical scavenging activity. The total phenolic compound (TPC) and the total flavonoid content were determined by the Folin–Ciocalteu method and colorimetric method, respectively. The phenolic compounds were identified and quantified by high-performance liquid chromatography (HPLC) coupled with an electrospray ionization mass spectrometry (ESI-MS). The results showed that *O. stricta* fruits present the best antioxidant activities than the two forms of *O. ficus indica*, while the TPC was more important in *O. ficus indica* than in the *O. stricta* fruits. The peels have higher flavonoids than pulp, and the thornless variety has more flavonoid than the spiny. The RP-HPLC and ESI-MS analysis detected two classes of phenolic compounds and betalain pigments. Isorhamnetin derivatives are the dominant flavonol glycoside identified in *O. ficus indica* (spiny: 65.25 μg·g^−1^; thornless: 77.03 μg·g^−1^) and *O. stricta* peels (19.22 μg·g^−1^).

## 1. Introduction

Several species of cactus pear plants belonging to the *Cactaceae* family originated from Central America (Mexico). These species became widespread in semi-arid regions of the world, including Tunisia [1]. They are of special interest because they are one of the few crops that can be cultivated in areas which offer very little growth possibility for common fruits and vegetables [2].

Cactus pear fruits have much commercial value. They are highly flavored, and have excellent nutritional properties. The fruits are used for the manufacture of food products such as juices [3,4], alcoholic beverages [5], jams [6] and natural liquid sweeteners [7]. One of the more valuable contributions of cactus fresh fruits to a diet is their vitamin C content. In addition to their nutritional properties, these plants contain biocompounds with several commercial applications. One such compound is betalain, a water-soluble nitrogen-containing pigment. It is found in high concentrations in cactus pear plants [8,9] in the form of betalamic acid, which is the chromophore common to all betalain pigments [10] used as natural food colorants [11,12,13]. The most important biocoumpounds in cactus fruit are phenolic compounds [14], betacyanins, and betaxanthin pigments, all of which have potent antioxidant properties [15,16]. Phenolic compounds can be defined as substances possessing an aromatic ring, carrying one or more hydroxyl groups, including their functional derivatives [17]. The chemical structure and concentration are quite variable and depend on the variety, ripening stages, and the kind of plant tissue [18]. Glycosilated flavonols, dihydroflavonols, flavonones and flavonols have been found in *Cactaceae* plants and fruits [19]. The antioxidant properties of the phenolic compounds in cactus pear plants makes the fruit an important product for preventing human health against degenerative diseases such as cancer, diabetes, hypercholesterolemia, arteriosclerosis or cardiovascular and gastric diseases, as is reported about other flavonol-rich foods [20,21,22,23]. The antioxidant activity of cactus fruit is twice as high as pears, apples, tomatoes, bananas, white grapes, and is comparable to red grapes and grapefruit [24]. Kuti [25] has reported an antioxidant effect due to the major flavonoids encountered in cactus fruits. Flavonoids are more efficient antioxidants than vitamins, since phenolic compounds are able to delay pro-oxidative effects in proteins, DNA, and lipids by the generation of stable radicals [26]. Polyphenolic compounds in *O. ficus indica* have been shown to induce a hyperpolarization of the plasma membrane and to raise the intracellular pool of calcium in human Jurkat T-cell lines [27].

Most of the studies made on cactus pear fruits consist of the chemical analysis of (a) the pulp for vitamin C, polyphenols, betalains and volatile constituent [28]; (b) the skin for polyphenols and lipids; and (c) the seeds for lipid profiles [29]. However, little is done to compare the main biocompounds in different species of the cactus pear fruit.

The most abundant species of cactus pear plants in Tunisia are the *O. ficus indica* and the *O*. *stricta* belonging to the orders *Centrospermae*, the genus *Opuntia* and subgenus *Platyopuntia* [30]. The *O. ficus indica* fruit, usually eaten fresh and after peeling, varies in shape, size and color (length: 30–65 mm, width: 20–40 mm), and consist of a thick peel and an edible juicy pulp with many hard seeds. The *O. stricta* ripe fruits are much smaller and not suitable for consumption (length: 20–50 mm, width: 15–30 mm). They have a red purple colored pericarp (thick peel) and a fleshy dark purple colored endocarp that contains small yellow and brown seeds embedded in pulp [31].

The aim of the present study is to provide new findings about biocoumpounds of the methanol extract of the fruits (peel and pulp) of the Tunisian *O. ficus* *indica* and *Opuntia stricta*. Previous studies have shown that the *Opuntia* species have a regional specificity. For example, some phenolic compounds have been identified in Tunisian *O. ficus indica* flowers and not exist in *O. ficus indica* flower cultivated in Sicily (Italy). De Leo *et al*. [32] analyzed the methanolic extracts of *O. ficus indica* flowers grown in Italy, the resulting chromatogram, showing nine peaks, only seven of them were identified as flavonol glycosylated derivatives. The results of Yeddes *et al*. [33] provided further information on phenolic acids and revealed even more peaks in the flavonol glycoside region chromatogram (eight flavonols were identified).

In keeping with a previous study on chemical composition, this present study reports a comparison of antioxidant activity and polyphenol and flavonoid content between the small red-purple fruits of *O. stricta* and the *O. ficus indica* fruits, followed by an investigation of the flavonol and betalain profile of Tunisian *Opuntia*.

## 2. Experimental

### 2.1. Plant Material

Fresh and mature pears fruits of two *Opuntia* species, two forms of *O. ficus indica*: spiny (green-yellow peel and yellow pulp) and thornless (green peel and red-purple pulp), and *O. stricta* (purple peel and pulp) were collected in summer 2011 (September). The spiny wild form was from the region of Al-Ala in the center of Tunisia, located at 35°36′N (North) latitude, 9°34′E (East) longitude, and 450 m (meter) altitude. The thornless cultivated form was from pilot cultivar of the Bou Argoub region in the northeast of Tunisia, located at 36°32′N latitude, 10°33′E longitude, and 62 m altitude. *O. stricta* was from a botanical garden in Tunis located at 36°49′N latitude, 10°11′E longitude, and 5 m altitude.

### 2.2. Solvents and Standard Phenolics

Methanol (CH_4_O), acetonitrile (CH_3_CN), formic acid (CH_2_O_2_) and acetic acid (CH_3_COOH) requested for chromatographic analysis and Folin Ciocalteu reagent were purchased from Merck Company (Merck, Darmstadt, Germany) and DPPH from Sigma Aldrich (St. Louis, MO, USA). Water was purified on a MilliQ system (Millipore S.A., Molsheim, France). The used standards were provided by Extrasynthese S.A. (Lyon, France) and were the following: rutin (quercetin 3-*O*-rutinoside), myricitrin (myricetin 3-*O*-rhamnoside), hyperoside (quercetin 3-*O*-galactoside), kaempferol 3-*O*-rutinoside, isorhamnetin 3-*O*-rutinoside, kaempferide (4′-methylkaempferol), rhamnetin (7-methylquercetin), isorhamnetin (3-methylquercetin), quercitrin (quercetin 3-*O*-rhamnoside) and myricetin.

### 2.3. Sample Preparation

*Opuntia* fruits were washed with distilled water, air-dried, and hand-peeled. Both, peel and pulp were freeze-dried and reduced into powders. The ground dried sample (30 mg) was extracted three times with 1.2 mL MeOH:acetic acid (99:1), sonicated in a water bath at room temperature for 15 min and then centrifuged at 3900 rpm for 15 min (Fish Bioblock Scientific). The argon is used to degas the combined supernatants. The mixture was immediately filtered through polytetrafluoroethylene (PTFE) membrane (0.45 μm) and stored in refrigerator at −30 °C. The described procedure is recommended for the total phenolic assay, total flavonoid assay, and RP-HPLC and ESI-MS analysis [34].

### 2.4. DPPH Radical Scavenging Activity Assay

The antioxidant capacity of the methanol extracts was tested by DPPH (1,1-diphenyl-2-picrylhyydrazyl) according to method adopted by Yen and Duth [35]. The DPPH method is the most used for the evaluation of the antioxidant properties and antiradical activity of natural products. DPPH is a stable free radical in a methanolic solution. An aliquot of the extracts above (10 μL, 20 μL, 40 μL, 80 μL, 180 μL, 380 μL, 580 μL, 780 μL, 980 μL, 1180 μL, 1580 μL, 1980 μL, 2380 μL, 2980 μL, 3780 μL) was mixed with 2 mL of DPPH, solution of varying concentrations were obtained. The mixtures were vortexed vigorously for 30 s and then immediately placed in an UV-visible lambda 25 model “Perkin Elmer” spectrophotometer. Scans of the solution were performed at wavelength ranging from 400 to 800 nm. The maximum absorbance was then read at 515 nm. When DPPH is placed in an antioxidant solution, its free radical is inhibited by the antioxidant before an absorbance measurement is performed. The inhibition of free radical DPPH (I%) was calculated as

*I*% = [(*A*_0_−*A*_1_)/*A*_0_] × 100 (1)

where A_0_ and A_1_ are the absorbance values of the blank (all reagents except the test compounds) and of the tested samples, respectively. The I% were plotted against respective concentrations used. The slope of the linear portion of each graph was used to calculate IC_50_% which is the concentration when 50% of the antioxidant is reduced. IC_50_% values of the extracts were compared to the IC_50_% value of a standard antioxidant, Trolox (6-hydroxy-2,5,7,8-tetramethylchroman-2-carboxylic acid) used as positive control and obtained by the same procedure.

### 2.5. Determination of the Total Phenolic Content

The amount of total polyphenolic compounds was determined according to Folin–Ciocalteu and adapted from Singleton and Rossi [36]. A 0.1 mL amount of methanol extracts was diluted to 0.5 mL with 2.5% (v/v) acetic acid. A 0.25 mL amount of Folin–Ciocalteu reagent (Merck) was added to 0.5 mL of the diluted extract and was allowed to stand for 3 min at room temperature. One mL of 200 g·L^−1^ Na_2_CO_3_ solution was added, and the volume was adjusted to 5 mL with distilled water. The mixture was then heated at 70 °C for 10 min. After cooling and color development, the absorbance was measured at 700 nm with a spectrophotometer UV (Spectrometer Spectra Max 384 PLUS Molecular Devices) using blank samples composed of distilled water and reagents. The amount of polyphenolic compounds is determined by comparing absorbance values of the samples to the absorption values of rutin and gallic acid (GA) (mg rutin or GA·g^−1^ fresh weight) standards.

### 2.6. Determination of Total Flavonoid Content

Total flavonoid content in the methanol extract was determined by using the colorimetric method (trichloride aluminum method) adopted by Bahorun *et al*. [37] in *Nigella sativa* extracts with slight modifications. An aliquot of methanol extracts (0.3 mL) was added to a 5 mL volumetric flask, containing 0.45 mL of distilled deionized water. After 5 min, 0.75 mL of 2% aluminum chloride (AlCl_3_·H_2_O) solution was added. The mixture was shaken and allowed to rest for 10 min of reaction. The absorbance was measured at 415 nm *versus* prepared methanol blank with an UV-VIS spectrophotometer (Spectrometer Spectra Max 384 PLUS Molecular Devices). The concentration of flavonoids was determined by comparing absorbance values of the sample to the absorption values of rutin used as a standard. Results were expressed as equivalent rutin (mg rutin·g^−1^of fresh weight) ± SD (standard deviation) with 3 replications.

### 2.7. RP-HPLC and ESI-MS Analysis

RP-HPLC analyses were performed using a Surveyor AS Autosampler including a binary Agilent HP 1100 pumping system, a thermostated column oven and a thermostated automatic injection module. The system was coupled to a TSP UV6000 detector UV used in 240–600 nm range. The column (Merck) was a reversed phase column (150 × 2.1 mm i.d., 3.5 μm, Agilent Eclipse XDB-RP). The injected volume was 4 μL and the column was thermostated at 30 °C. The elution solvent was a mixture of solvent A, consisting of ultrapure water/formic acid (99.9:0.1, v/v) and solvent B, consisting of acetonitrile/formic acid (99.9:0.1, v/v), and the following gradient was applied: initial, 3% B; 0–5 min, 9% B linear; 5–15 min, 16% B linear; 16–45 min, 50% B linear; the gradient was followed by washing and reconditioning of the column. Helium was used for degassing solvents. Two particular wavelengths were used for quantification of polyphenols: 280 nm for phenolic acids, 350 nm for flavonols [30]. Quantitative determination was carried out using calibration curves of standards. Phloretic acid and hyperoside were chosen as external standards for quantification of phenolic acids, and flavonols. ESI-MS analyses were performed in negative mode for phenolic compounds (molecular fragmentation losses hydrogen). For better identification of the peaks, the positive mode was used for betalains (molecular fragmentation with hydrogen acquisition). These tests were performed on an LCQ DECA ion trap mass spectrometer (Thermo-Finnigan, San José, CA, USA) equipped with an ESI source and run by Xcalibur (version 1.2) software. The parameters were as follows: ion spry voltage, 3.69 kV; capillary voltage, −70.78 V; capillary temperature, 240.4 °C; sheath nitrogen gas flow rate, 66.65 (arbitrary units); auxiliary gas flow rate, 3.81 (arbitrary units); scan range of *m/z* 50–2000. Samples corresponding to collected HPLC peaks were directly introduced into the ESI source by a built-in syringe pump at 3 μL. For the generation of MS^n^ data, the precursor ions were fragmented by helium gas collision in the ion trap by optimizing the collision energy in order to obtain the intensity of the precursor ion close to 10% of the relative scale spectrum.

### 2.8. Statistical Analysis

All experiments were the result of three runs that averaged together. The standard deviations were based on triplicate measurements (*n* = 3). The value for each sample was expressed as the mean (M) ± standard deviation (SD). The analyses of variance were performed by ANOVA with software SPSS version 11.5 for Windows [38]. Differences among the means were compared using the Fisher–Snedecor distribution with a level of significance *p* < 0.05.

## 3. Results and Discussion

### 3.1. DPPH Radical Scavenging Activity

The antioxidant activity of Tunisian *O. stricta* and *O. ficus indica* fruit methanol extracts of both peel and pulp are compared and shown in Table 1. Results show that there is little difference in the antioxidant activity between the spiny and the thornless *O. ficus indica* (for both peel and pulp). The antioxidant activity of the *O. stricta* and *O. ficus indica* are lower compared to trolox (for both peel and pulp). Results also show that the peel extract for the *O. stricta* has a higher antioxidant activity than the pulp (about 3% higher). However, for the *O. ficus indica*, the pulp has a higher antioxidant activity than the peel (about 2%). The average antioxidant activity of the peel and the pulp of the *O. stricta* is higher than the average antioxidant activity for *O. ficus indica* (about 13% higher). Statistically, the methanol extract from Tunisian *Opuntia* fruits have similar reductive ability (*F*_2,6_ = 5.14, *p* < 0.05; Table 1).

**Table 1:** DPPH antioxidant scavenging capacity (IC_50_%) of two Tunisian *O. ficus indica* forms and *O. stricta* fruit methanol extract and Trolox.

		DPPH Antioxidant Scavenging Capacity IC_50_% (mg·mL^−1^) ^a^	
	Tissue Fruit	Peel	Pulp
Species-Forms			
*O. ficus indica* “spiny”		0.54 ^b^ ± 0.04	0.51 ^c^ ± 0.01
*O. ficus indica* “thornless”		0.57 ^b^ ± 0.02	0.56 ^c^ ± 0.01
*O. stricta*		0.40 ^b^ ± 0.03	0.43 ^c^ ± 0.01
Trolox		0.33 ± 0.01	

^a^ Means of triplicate assays (mg·mL^−1^) ± SD. ^b, c^ Means of triplicates in the same column with same letters indicate no significant difference at values p < 0.05.

Our results are in contradiction with the results of Moussa-Ayoub *et al*. [39], who used the electron paramagnetic resonance spectroscopy to determine the antioxidant activity. Moussa-Ayoub *et al*. [39] showed that the *O. ficus indica* (from Sicily/Italy) peel has an antioxidant activity higher than the pulp (about a 35% discrepancy). They explained their results by the presence of large amounts of flavonols, phenolics, as well as betacyanins in the fruit’s peel of *O. ficus indica* compared to its pulp. Maataoui *et al*. [40] showed that the purple juice of the *O. ficus indica* has a higher antioxidant activity than the yellow-orange juice. Ammar *et al*. [41] studied flower extracts at the post-flowering stage for both *O. stricta* and *O. ficus indica*. Their results show that *O. stricta* has a higher antioxidant activity than *O. ficus indica*. All these results indicate that *O. stricta* has higher antioxidant activity than *O. ficus indica*, regardless of the part of fruit studied. This is possibly related to the darker color of the flower and the fruit.

### 3.2. Total Phenolic Contents

**Figure 1:**
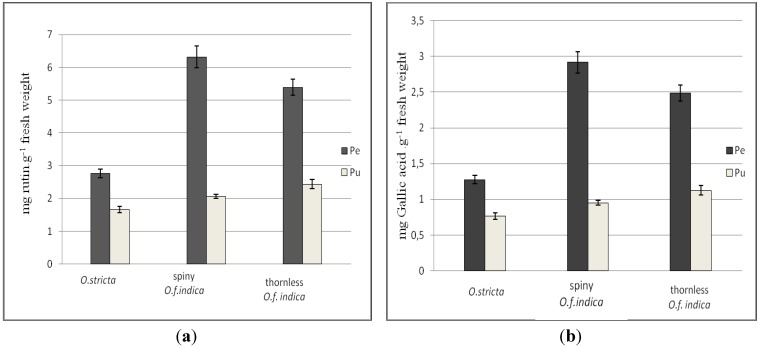
Total phenolic content of peel (Pe) and pulp (Pu) of three Tunisian *Opuntia* (*O*.) forms. Results are expressed as mg rutin equivalents g^−1^ fresh weight (**a**) and as mg GA equivalents g^−1^ fresh weight (**b**) (mean ± SD, *n* = 3). Means of triplicates pulp and pulp indicate no significant difference (level of significance *p* < 0.05) between the three Tunisian *Opuntia* forms.

Polyphenols are an important group of natural compounds, recently considered to be of high scientific and therapeutic interest. The total polyphenols content (TPC) was estimated in different methanol extracts (Figure 1). Results show that the amounts of polyphenols were much greater in the peel than in the pulp. The TPC in the peel of the spiny *O. ficus indica* was three-fold higher than the pulp. The TPC in the peel of the thornless was 2.21-fold higher than the pulp. Similar results were observed for the *O. stricta*, for which the peel was 1.66-fold higher than the pulp. The comparison between the two cactus pear species shows that the average TPC in the *O. ficus indica* is higher than the average TPC in the *O. sticta* (1.94-fold higher for spiny form, and 1.76-fold higher for the thornless form). The total phenolic contents were not statistically different among the various forms of Tunisian *Opuntia* fruits (*p* < 0.05). In conclusion, results show that the TPC is higher in the peel than in the pulp and is also higher in the *O. ficus indica* than the *O. stricta*. These results are in agreement with the work of Moussa Ayoub *et al*. [39] and Díaz Medina *et al*. [42]. All of which showed that the highest concentrations of phenolic compounds in fruits occurred in the skin tissue.

### 3.3. Total Flavonoid Content

Our work shows that there are much less flavonoids than phenolic compounds in all the cactus pear species studied. The total flavonoid content is expressed as a concentration in mg of equivalent rutin g^−1^ FW. Results show that the flavonoid content in the peel of the *O. ficus indica* is higher than in the pulp (4-fold higher for the thornless and 2.30-fold higher for the spiny). For the *O. stricta*, the total flavonoid content in the peel is 1.42-fold higher than the pulp. The total flavonoid content of the thornless *O. ficus indica* is higher than the total flavonoid content of the *O. stricta* (1.47-fold higher). The total flavonoid is higher in the thornless *O. ficus indica* than the spiny (1.76-fold higher). These differences were statistically non-significant (*p* < 0.05). In conclusion, the peel contained more flavonoids than the pulp; the thornless contained more flavonoids than the spiny, and the *O. ficus indica* has more flavonoids than the *O. stricta*. These results are in agreement with the work of Ndhlala *et* *al*. [43].

**Figure 2:**
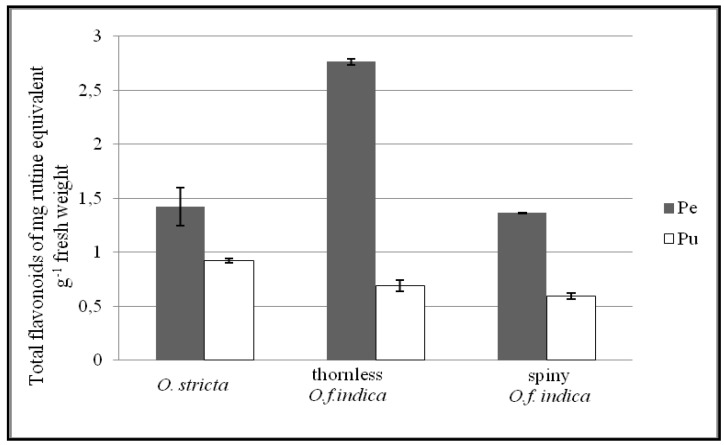
Total flavonoid content of peel (Pe) and pulp (Pu) of three Tunisian *Opuntia* (*O.*) forms. Results are expressed as mg rutin equivalents g^−1^ fresh weight (mean ± SD, *n* = 3)*.* Means of triplicates pulp and pulp indicate no significant difference (level of significance *p* < 0.05) between the three Tunisian *Opuntia* forms.

**Figure 3:**
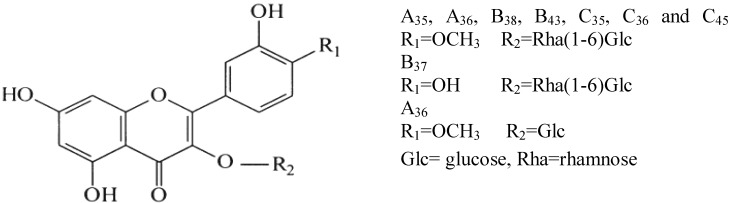
Chemical structures of flavonol glycosides found in methanol extract fruits of Tunisian *O. ficus indica* and *O. stricta*.

It is known that large amounts of polyphenols and flavonoids increase antioxidant activity. Polyphenols are endowed with potent antioxidant activities *in vitro*, but *in vivo*, the scantiness of biomarkers was the major limitation by the lack of robust biomarkers [44]. The monofloral Cuban honey analysis showed high concentrations of phenolic acids and flavonoids, which are responsible, at least in part, for their antioxidant activity [45]. However, this is contrary to what was found in this work. In this study, we observed in the pulp of the *O. ficus indica*, a higher antioxidant activity and lower content of polyphenols and flavonoids. (Table 1, Figure 2, Figure 3). Recent studies on the antioxidant activity of *O. ficus indica* and *O. stricta* flowers reported by Ammar *et al*. [41] showed that, during the initial flowering stage, the *O. stricta* contains a low amount of total phenolics and a high antioxidant activity. The antioxidant capacity is determined not only by concentration, but also by several other factors such as the reactivity toward radicals, and the distribution, localization, and fate of antioxidant-derived radicals in interaction with other antioxidants [46]. In order to evaluate the antioxidant capacity, these factors should be separately assessed and considered. We can attribute the antioxidant activity of the pulp to other compounds and explain our results in comparison to Niki’s studies on antioxidant capacity [46]. According to the literature, the content of vitamin C and betalains in *Opuntia* pulp fruits provided higher antioxidant activity and offered a preventive potential against oxidative stress in the human body. Many investigations reported the non-negligible antioxidant activity of betalains due to their redox potentials. Several works have demonstrated the potent antiradical scavenging activity of betalains *in vitro* [47]. In conclusion, the antioxidant activity as shown in this work is related to more than the amount of polyphenols and flavonoids.

### 3.4. RP-HPLC and ESI-MS Analysis

Very little work is available in the literature on the presence of flavonols in the *O. stricta* fruit. In this investigation, we identify and compare amounts of flavonols and other bioactive compounds in the peel and pulp of the Tunisian *O. ficus indica* and the *O. stricta*. The method used in this work is RP-HPLC coupled with UV and ESI-MS. The identification of the compounds (Table 2) was based on a comparison of their HPLC retention times (RT), UV-visible spectrum, mass spectrometry (MS and MS^2^ fragments) with authentic standards compounds and published data. The betacyanins peaks were identified by order of elution, spectral characteristics, and molecular ions [M + H]^+^ with the red beet methanol extract injected in the same conditions as the samples. According to the available standards, we identified nine chromatographic peaks: A_35_, A_36_, B_38_, B_43_, C_35_, C_36_ and C_45_ as isorhamnetin-3-*O*-rutinoside, A_36_ as isorhamnetin-3-*O*-glucoside and B_37_ as quercetin-3-*O*-rutinoside (Figure 3). All the identified flavonoids belong to the group of flavonol aglycones. Table 2 also shows the quantification of phenolic compounds.

Three types of compounds were detected by UV: two classes of phenolic compounds (flavonols detected at 350 nm and phenolic acids detected at 280 and 320 nm) and betalain pigments detected at 540 nm and 470 nm (Table 2).

**Table 2:** RT, Spectral, Characteristic ions (MS and MS^2^) and main amount compounds data from pulp and peel methanol extract of Tunisian *O. ficus indica* and *O. stricta*.

Tunisian *Opuntia*	Tissues	Major Peaks	Bioactive Compounds	RT (min)	MS (*m/z*)	MS^2^ (*m/z*)	λ_max_ (mn)	Amount (μg·g^−1^ Fresh Weight) M ± SD
Spiny *O. ficus indica*	Peel	A_31_	unidentified flavonol	18.45	741	300	352	3.13 ± 0.04
		A_32_	Isorhamnetin diglycoside	20.68	769	314–605	355	14.60 ± 0.009
		A_33_	Isorhamnetin diglycoside	21.05	755	315–605	354	45.59 ± 0.01
		A_34_	unidentified flavonol	22.43	609	315–314	263–353	17.48 ± 0.07
		A_35_	Isorhamnetin-3- *O*-rutinoside	23.75	623	315–623	352	2 ± 0.03
		A_36_	Isorhamnetin-3- *O*-rutinoside	24.03	623	315–623	352	1.98 ± 0.002
		A_37_	Isorhamnetin-3- *O*-glucoside	24.93	477	314–477	352	1.08 ± 0.01
		A_38_	phenolic acid	28.68	431	193–236	328	1.81 ± 0.02
		A_51_	Indicaxanthin	7.22	309	265	485	n.q
	Pulp	A_61_	Indicaxanthin	6.98	309	239	485	n.q
Thornless *O. ficus indica*	Peel	B_31_	phenolic acid	12.55	239	239	328	2.44 ± 0.06
		B_32_	phenolic acid	14	355	193–355	326	4.69 ± 0.02
		B_33_	phenolic acid	15.53	489	235–193	325	8.64 ± 0.03
		B_34_	Isorhamnetin diglycoside	20.58	769	315–605	355	13.07 ± 0.05
		B_35_	Isorhamnetin diglycoside	20.95	755	315–605	354	35.09 ± 0.01
		B_36_	Isorhamnetin diglycoside	21.2	755	315–605	353	10.13 ± 0.02
		B_37_	quercetine-3- *O*-rutinoside	22.35	609	301	350	5.60 ± 0.05
		B_38_	Isorhamnetin-3- *O*-rutinoside	23.97	623	315	354	18.74 ± 0.08
		B_39_	phenolic acid	28.65	431	193–237	326	5.43 ± 0.04
		B_51_	Betanin (betanidin-5- *O*-β-glucoside)	9.52	551	475–312	530	n.q
	Pulp	B_41_	phenolic acid	20.6	613	562–477	270–332	0.34 ± 0.001
		B_42_	phenolic acid	20.97	565	339	325	0.75 ± 0.003
		B_43_	Isorhamnetin3- *O*-rutinoside	23.97	623	315	355	0.39 ± 0.005
		B_61_	Betanin	7.43	551	389	537	n.q
		B_62_	Isobetanin (isobetanidin-5- *O*-β-glucoside)	8.43	551	389	537	n.q
		B_63_	betanidin	9.63	389	389	541	n.q
*O. stricta*	Peel	C_31_	phenolic acid	12.57	395	349	325	4.15 ± 0.002
		C_32_	unidentified compound	16.2	977	815	346	n.q
		C_33_	unidentified compound	18.68	639	477	346	n.q
		C_34_	unidentified compound	19.27	611	431	345	n.q
		C_35_	Isorhamnetin-3- *O*-rutinoside	23.68	623	315	352	4.95 ± 0.01
		C_36_	Isorhamnetin-3- *O*-rutinoside	23.97	623	315	355	14.27 ± 0.02
		C_51_	Betanin	7.72	551	389	533	n.q
		C_52_	Isobetanin	8.58	551	389	533	n.q
		C_53_	betanidin	9.78	389	343–150	539	n.q
	Pulp	C_41_	phenolic acid	12.62	395	395–349	326	0.45 ± 0.003
		C_42_	phenolic acid	16.08	977	815	330	0.41 ± 0.01
		C_43_	phenolic acid	18.58	639	477	330	0.43 ± 0.02
		C_44_	phenolic acid	19.17	611	431	334	0.43 ± 0.009
		C_45_	Isorhamnetin-3- *O*-rutinoside	23.68	623	315	350	0.85 ± 0.01
		C_46_	Phenolic acid	24.87	477	314	331	2 ± 0.02
		C_61_	Betanin	7.53	551	389	538	n.q
		C_62_	Isobetanin	8.4	551	389	533	n.q
		C_63_	betanidin	9.87	389	343	525	n.q

M, values expressed as means (μg·g^−1^) ± SD, standard deviation. n.q, not quantified but identified. Each sample was analyzed in triplicate.

(a) Flavonol glycosyl: The flavonol glycosil class was indicated by absorbance at 255–263 nm and 350–355 nm in accordance with the work of Wollenweber [48]. He analyzed the flavonoids in the fronds of *Cheilanthes farinosa* (Polypodiaceae) and detected such peaks at the mentioned wavelength. Results show that the peel of the thornless *O. ficus indica* has the highest amount of phenolic compounds. The most dominant flavonol glycosil are isorhamnetin derivatives. They are found in the peels but not in the pulp (very small amount of isorhamnetin-3-*O*-rutinoside in the thornless forms and *O. stricta*). The content of these compounds are as follows: thornless *O. ficus indica*: 77.03 μg·g^−1^ spiny *O. ficus indica*: 65.25 μg·g^−1^; and *O. stricta* 19.22 μg·g^−1^. The peel also contained a compound not found in the spiny *O. ficus indica* and the *O. stricta*. This compound was identified as quercetin 3-*O*-rutinoside (5.60 ± 0.05 μg·g^−1^ FW). Compared to Tunisian fruits that contained more isorhamnetin, less quercetin and no kaempferol, Kuti [14] showed that the Texas fruit has more quercetin, less isorhamnetin, and some kaempferol. Fernández-López *et al*. [49] reported for *Opuntia* fruits significant amounts of flavonoids, being quercetin in the predominant type followed by isorhamnetin, luteolin and kaempferol. These results indicate that the phenols are region-specific. Our MS results showed a signal at *m/z* 769 (A_32_ and B_34_). The corresponding MS^2^ spectrum exhibited a main product ion at *m/z* 605 and 314. These results could be correlated to the presence of isorhamnetin [28]. The signal at *m/z* 605 corresponds to the loss of a fragment with −146 amu (atomic mass unit) in accordance with the mass of a deoxyhexose moiety flowed by one water molecule ([M − H − 146 − 18]^−^). The fragment at 314 corresponds to the molar mass of an ionic isorhamnetin. The MS spectrum of peaks A_33_, B_35_ and B_36_ produced a pseudomolecular ion [M − H]^−^ at *m/z* 755, releasing a major MS^2^ fragment at *m/z* 605 = ([M − H −132 − 18]^−^) and 315, −132 amu is the molar mass of a pentose which loses a water molecule, thus allowing us to hypothesize that those signals correspond to an isorhamnetin diglycoside containing one pentose molecule. All these MS results prove the presence of isorhamnetin derivatives.
(b) Phenolic acids: The highest content of total phenolic acids was found in thornless *O. ficus indica* peel (21.2 μg·g^−1^ FW) followed by *O. stricta* peel (4.15 μg·g^−1^ FW) and pulp (3.72 μg·g^−1^ FW). No phenolic acid was detected in the spiny *O. ficus indica* pulp.
(c) Betalain: The A_61_ showed a pseudomolecular ion [M + H]^+^ at *m/z* 309. The corresponding MS^2^ spectrum exhibited a main product ion at *m/z* 239. The A_61_ spectrum (MS and MS^2^) was indicative of indicaxanthin. The MS spectrum of peaks B_51_, B_61_, C_51_, and C_61_ produced a pseudomolecular ion [M + H]^+^ at *m/z* 551 and the MS^2^ spectrum showed produced ions at *m/z* 389. The peaks were identified as betanin. The chromatographic profile of betalain peel showed the richness of the spiny *O. ficus indica* in indicaxanthin (A_51_ at 470 nm) and the *O. stricta* in betacyanin (C_51_, C_52_ and C_53_ at 540 nm). Table 2 revealed a wealth in betacyanin in *O. stricta* and thornless *O. ficus indica* pulp. Our results are in agreement with the results of Castellar *et al*. [31], who found that the level of betanin and isobetanin were around five times higher in Murcia *O. stricta* (southeast of Spain) than in the red-purple fruits of *O. ficus indica*.

## 4. Conclusions

This comparative study of Tunisian *O. ficus indica* and *O. stricta* fruit peel and pulp indicated the presence of biocompounds with possible commercial applications. These biocompounds include polyphenol, and betalains, all of which have antioxidant activity. The chemical composition, the amounts and the nature of compounds vary with species and forms (spiny and thornless). Thus have different antioxidant activity. The *O. stricta* has higher antioxidant activity than the *O. ficus indica*. The peel has higher antioxidant activity than the pulp. The high antioxidant activity of the *O. stricta* is related to the high level of betalain pigments, whereas the high antioxidant activity in the peel is related to the high level of TPC and flavonoids. The main polyphenols in *O. ficus indica* peel were flavonols, more precisely isorahamnetin derivatives. The main polyphenols in the *O. stricta* pulp are phenolic acids. The quercetin 3-*O*-rutinoside was only found in the spiny *O. ficus indica*. These findings make Tunisian *Opuntia* fruits a promising source of biologically active polyphenolic and betacyanins mixtures. Further investigations need to be done to identify the unknown peaks and compounds. One suggestion would be to compare these peaks to those produced by known standards using other techniques of extraction and identification. This work can be repeated with samples of the cactus fruits collected at different Tunisian regions and seasons to see how the climate and soil affect the results. Another approach would be to change the methods of extractions with different solvents and compositions.
